# A fully automatic cardiac model with integrated scar tissue information for improved assessment of viability

**DOI:** 10.1186/1532-429X-14-S1-M12

**Published:** 2012-02-01

**Authors:** Mirja Neizel, Yang Chul Boering, Florian Bönner, Jan Balzer, Malte Kelm, Burkhard Sievers

**Affiliations:** 1 Department of Cardiology, University Hospital Düsseldorf, Düsseldorf, Germany

## Summary

This study evaluates whether integration of scar tissue into a patient specific heart model derived from Magnetic Resonance images is feasible.

## Background

Treatment of coronary artery disease can only be effective if myocardial tissue can be identified, that is still viable. To localize scar tissue in relation to the coronary vessels would therefore be desirable for interventional guidance. The aim of this study was to evaluate 1) whether integration of scar tissue into a patient specific heart model derived from Magnetic Resonance (MR) images is feasible and 2) whether this patient specific heart model allows for accurate assessment of the amount of myocardial scar compared to conventional manual infarct sizing.

## Methods

20 patients with clinical indication for viability testing (EF 47±13%) were investigated on a 1.5 Tesla MRI (Achieva, Philips, The Netherlands). Images using a free-breathing 3D-wholeheart balanced TFE sequence triggered to end-diastole were acquired. 10 minutes after injection of a contrast agent (0.2 ml/kg/body weight Magnevist®, Bayer, Germany) delayed enhancement (DE) images were acquired using a 3D inversion recovery sequence. Fully automatic segmentation of the heart and great vessels was performed using a comprehensive surface model (Philips Research Laboratories, Aachen, Germany). In addition, information of scar tissue was merged fully automatically from 3D-DE sequence into the segmented whole heart model. Finally, an automatic signal intensity threshold for scar identification was determined. Infarct size and end diastolic left ventricular (ED-LV) volume derived from this model were compared to conventional manual assessment of infarct size and LV volume.

## Results

11 Patients had a chronic occlusion of the right coronary artery (RCA), 3 patients of the left circumflex (LCX) and 6 patients of the left anterior descending artery (LAD).

Integration of viability information into a patient specific heart model was feasible in all cases with identical location of scar tissue compared to DE images.

Infarct size averaged 19±15 g by manual tracing and 15±14 g by automatic segmentation (Concordance correlation coefficient 0.94; limits of agreement ±7%). Location of scar tissue was identical between the two analysis methods for all cases.

ED-LV volume was 151±14ml by manual assessment and 153±16ml by automatic segmentation (Concordance correlation coefficient 0.65; limits of agreement ±25%).

## Conclusions

A cardiac model with integrated scar tissue information derived fully-automatically from a 3D whole heart MR acquisition as well as a 3D DE-data set is feasible and allows for precise localization and determination of the amount of myocardial scar.

## Funding

No funding.

**Figure 1:**
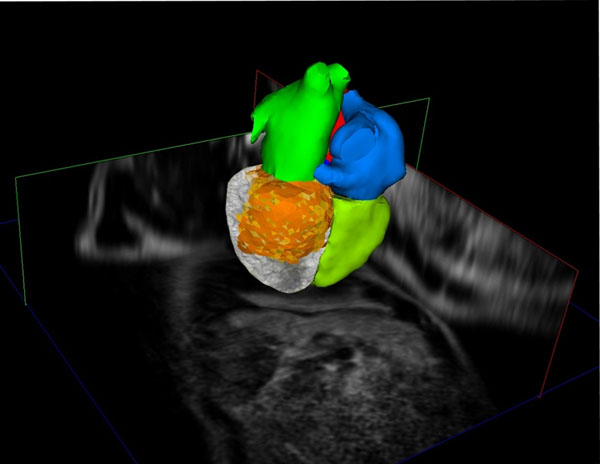
Fully automatic segmentation of a whole heart 3D MR data set. Using the common patient coordinate system an approximate registration of the adapted model with 3D late enhancement data can be achieved. Orange colour indicates scar tissue.

